# The Relationship Between Gestational Diabetes, Emotional Eating, and Clinical Indicators

**DOI:** 10.3390/medicina61081447

**Published:** 2025-08-12

**Authors:** Tuğçe Taşar Yıldırım, Çiğdem Akçabay, Sevler Yıldız, Gülşen Kutluer

**Affiliations:** 1 Fethi Sekin Research and Training Hospital Internal Medicine Clinic, Elazıg 23000, Türkiye; 2 Fırat University Hospital Obstetrics and Gynecology Clinic, Elazıg 23000, Türkiye; cagcabay@hotmail.com; 3 Fethi Sekin Research and Training Hospital Psychiatry Clinic, Elazıg 23000, Türkiye; dr_sevler@hotmail.com; 4 Anadolu Hospital Obstetrics and Gynecology Clinic, Elazıg 23000, Türkiye; drgulsenkutluer23@gmail.com

**Keywords:** gestational diabetes mellitus, anxiety, depression, eating disorders

## Abstract

*Background and Objectives*: Gestational diabetes mellitus (GDM), which is becoming increasingly common in contemporary society, is recognized for its considerable psychosocial impact on pregnant women throughout the perinatal phase. The purpose of this research was to explore the possible links between mental health status and dietary habits among pregnant women diagnosed with GDM, alongside examining how these factors correlate with clinical indicators like HbA1c measurements and the necessity for insulin therapy. *Materials and Methods*: The study included 82 pregnant participants, 37 with gestational diabetes mellitus and 45 without. Blood samples were collected from all participants for biochemical analysis, including fasting blood glucose, postprandial blood glucose, and HbA1c levels, which can be clinical indicators for the presence of gestational diabetes mellitus, and the need for insulin treatment was recorded. Then, participants completed a questionnaire collecting sociodemographic and clinical data as well as the Beck Anxiety Inventory (BAI), Beck Depression Inventory (BDI), Salzburg Emotional Eating Scale (SEES), and REZZY Eating Disorders Scale (REZZY). Data were statistically analyzed. *Results:* A previous diagnosis of gestational diabetes was more frequent in the case group (18.9%) than in the control group (2.2%) (*p* = 0.020). OGTT positivity was detected in 56.8% of the case group, whereas all control participants had negative results (*p* < 0.001). There were no statistically significant differences between the two groups in psychological symptom scores or eating behavior assessments (*p* > 0.05). *Conclusions:* Pregnant women with gestational diabetes mellitus were observed to score higher on measures of anxiety, depression, and emotional eating, particularly in response to negative emotions. These findings may indicate a potential association between gestational diabetes and psychological or behavioral factors related to metabolic regulation during pregnancy.

## 1. Introduction

Gestational diabetes mellitus (GDM) is defined as impaired glucose regulation resulting in elevated blood sugar levels that are first detected during pregnancy in women without a previous diabetes diagnosis [1]. This condition represents nearly 86% of hyperglycemia cases occurring throughout pregnancy [2]. Compared to pregnant women with normal blood glucose levels, those diagnosed with GDM have a greater likelihood of experiencing complications for both mother and infant during pregnancy, as well as an increased risk of developing type 2 diabetes, cardiovascular conditions, lipid abnormalities, and various metabolic disorders later in life [3,4,5,6].

Multiple factors contribute to the risk of developing GDM, including older maternal age, obesity, previous diagnosis of polycystic ovary syndrome, and a family history of diabetes [7]. Diagnosis commonly occurs between the 24th and 28th weeks of pregnancy through the oral glucose tolerance test (OGTT). GDM is linked to higher complications during pregnancy, such as macro-somia, pre-eclampsia, and difficulties during labor, in addition to an increased risk of type 2 diabetes for the mother in the long term [8].

Management of GDM primarily involves medical nutrition therapy, with a focus on controlling blood glucose levels through regulated carbohydrate intake, regular glucose monitoring, and, when necessary, insulin therapy. However, adherence to strict dietary regimens during pregnancy can be a source of considerable psychosocial stress for some women [9]. Indeed, many pregnant women diagnosed with diabetes report feeling constant anxiety about food choices, often describing their relationship with food as obsessive—closely resembling patterns seen in eating disorders [10]. Furthermore, maintaining recommended dietary and weight gain guidelines can be particularly challenging for women with GDM. This difficulty may result in periods of binge eating or, conversely, extreme dietary restriction, both of which may negatively impact maternal and fetal health outcomes [11].

Pregnancy is a unique physiological state marked by profound psychological and biological changes, which can significantly alter body image perception and influence eating behaviors. As such, this period may serve as a potential trigger for the emergence or exacerbation of symptoms associated with eating behavior disorders [12]. During pregnancy, alterations in eating patterns are common and may include behaviors such as excessive food intake, strong aversions to specific foods or beverages, and shifts in taste perception. These pregnancy-induced bodily changes can, in some cases, precipitate disordered eating even in women without a prior history of such conditions [13,14].

Anxiety associated with changes in body shape, worry about weight gain, and dissatisfaction with pregnancy weight progression may be associated with maladaptive eating behaviors in pregnant women [15,16]. Moreover, neuroendocrine adaptations that occur as part of the physiological adjustments during pregnancy may affect brain function, leading to disruptions in metabolic processes, appetite regulation, and mood stability [17].

Research indicates that pregnant individuals with existing eating disorders are at elevated risk for pregnancy- and delivery-related complications, including hyperemesis gravidarum, prolonged labor, and an increased likelihood of cesarean delivery or labor induction. Additionally, disordered eating during pregnancy has been linked to a higher incidence of anemia, hypertensive disorders, and gestational diabetes [17,18,19]. Beyond eating disorders, psychological factors such as anxiety and depression—possibly influenced by physiological changes—are also considered to play a role in the onset of GDM [20]. Nevertheless, current research has yet to establish a clear consensus on this association. Some research suggests that anxiety and depression may cause the development of GDM through sustained activation of the hypothalamic–pituitary–adrenal (HPA) axis, leading to increased cortisol secretion and insulin resistance [21]. Additionally, there is evidence suggesting that a GDM diagnosis may heighten the risk of experiencing depression during pregnancy or after childbirth, pointing toward a potentially reciprocal relationship between GDM and mood disorders [22].

Conversely, other studies have found no significant association between anxiety or depression and the incidence of GDM, nor between a GDM diagnosis and the subsequent development of prenatal or postnatal depression [23,24,25,26]. These conflicting findings highlight the lack of consensus in the current literature and underscore the need for further research to clarify the potential psychological implications of GDM and its bidirectional interactions with mood disorders.

Emotional eating is often regarded as a coping strategy employed by individuals in response to negative emotional states [27]. This behavior may manifest as either increased or decreased food intake triggered by emotional stimuli, which can range from pleasurable to distressing experiences [28]. Pregnancy represents a period of profound hormonal, physiological, and psychosocial transformation in a woman’s life. During this period, pregnant women commonly experience both physical changes and increased emotional variability [29].

Several factors have been identified as contributing to emotional eating during pregnancy, including altered body image, limited social support, insufficient stress management skills, and concerns related to the pregnancy itself [30]. In light of these considerations, we hypothesized that stress, anxiety, depression, and hormonal fluctuations occurring during pregnancy may significantly influence maternal eating behaviors. Therefore, this study sought to examine the connections between anxiety, depression, disordered eating patterns, emotional eating, and the occurrence of GDM.

## 2. Materials and Methods

The study was carried out following the ethical guidelines set forth in the 2013 update of the Declaration of Helsinki. Approval for the research was granted by the Ethics Committee for Non-Interventional Clinical Studies at Fırat University (No. 2024/01–28, dated 9 January 2024).

This study was designed as an analytical, cross-sectional observational study. Female patients attending the Internal Medicine and Obstetrics and Gynecology outpatient clinics at Fırat University Hospital from December 2024 to February 2025 were recruited for the study. Participants were either newly diagnosed with GDM and had not yet initiated any treatment or were healthy pregnant women attending routine prenatal check-ups. Inclusion criteria were age between 18 and 45 years, being in the second or third trimester of a singleton pregnancy, and giving informed consent for participation. Exclusion criteria included a history of pregestational diabetes, previous diagnosis of psychiatric disorder, current use of psychotropic medication, history of eating disorders, or any systemic disease that could affect metabolic or psychological parameters. Participants who did not meet the inclusion criteria or had missing data from the questionnaire or laboratory assessments were excluded. As a result, 5 individuals were excluded from the initial sample due to missing data or meeting one or more exclusion criteria. The study participants did not include women with twin pregnancies or those conceived with assisted reproductive techniques (ART), as these are additional factors that may contribute to the development of GDM and depressive disorder. The control group was selected to match the GDM group in terms of sociodemographic characteristics. All participants in the control group were free from known systemic or psychiatric disorders.

GDM was diagnosed using an oral glucose tolerance test, following the American Diabetes Association’s recommendation for universal screening between 24 and 28 weeks of pregnancy [30]. Both the one-step and two-step diagnostic approaches were used in accordance with clinical practice.

In the two-step diagnostic approach, participants first underwent a 50 g oral glucose loading test, and a plasma glucose concentration ≥ 140 mg/dL at 1 h was considered a positive screening result. Women with a positive result proceeded to a diagnostic oral glucose tolerance test (OGTT) using 75 g (2 h) or 100 g (3 h) of glucose, depending on the health center’s clinical protocol. GDM was diagnosed if two or more plasma glucose values exceeded predefined thresholds on the 100 g OGTT or if one or more values were above the cut-off value on the 75 g OGTT. In the one-step approach, a 2 h 75 g OGTT was performed directly, and GDM was diagnosed if any of the measured values exceeded the established diagnostic criteria. The choice of diagnostic method was based on the clinical setting and reflects routine practice in our clinic rather than study-specific procedures.

Scales Utilized in the Study

Sociodemographic and Clinical Information Form:

A structured questionnaire was developed by the researchers based on clinical experience and the scientific literature. This form was used to collect demographic characteristics of the participants, clinical history (including pregnancies, duration of illness), and relevant biochemical parameters.

Beck Depression Inventory (BDI):

This scale is a widely used self-report instrument designed to assess the severity of depressive symptoms in adults. Total scores are interpreted as follows: 0–9 indicates minimal or no depression, 10–18 mild depression, 19–29 moderate depression, and 30–63 severe depression. The version of the scale adapted into Turkish by Hisli (1989) showed satisfactory psychometric properties [31,32,33].

Beck Anxiety Inventory (BAI):

This scale is a self-administered instrument developed by Beck et al. to assess the intensity of anxiety symptoms. Score interpretation is as follows: 8–15 indicates mild anxiety, 16–25 moderate anxiety, and 26–63 severe anxiety. Turkish adaptation and validation were performed by Ulusoy et al. [34,35].

Salzburg Emotional Eating Scale (SEES):

The Salzburg Emotional Eating Scale (SEES) is a 20-item questionnaire developed to evaluate emotional eating by measuring changes in food intake across various emotional states. The scale is divided into four subdomains, each with five items: positive emotions (e.g., happiness), low-arousal negative emotions (e.g., sadness), and high-arousal negative emotions (e.g., anger and anxiety). Participants respond using a 5-point Likert scale, where 1 indicates significantly reduced food intake and 5 reflects a substantial increase. A score above 3 suggests overeating in response to emotions, a score of 3 represents stable eating behavior, and scores below 3 reflect decreased consumption. High scores indicate a tendency to eat more when experiencing emotional distress. The Turkish adaptation of the SEES has undergone validation and reliability testing [36,37].

REZZY Eating Disorders Scale (SCOFF):

This 5-item screening tool, developed by Morgan et al. (1999) as the SCOFF questionnaire, identifies individuals at risk for eating disorders by assessing disordered eating behavior and body image concerns [37]. The Turkish version, renamed REZZY, retains the original format and scoring method. Each “yes” response is scored as 1 point; a total score of ≥2 indicates potential risk for an eating disorder [38,39].

Laboratory Samples

Biochemical analyses in this study were carried out at the central laboratory of our institution using the Beckman AU-5800 analyzer (Beckman Coulter Diagnostics, Indianapolis, IN, USA). The parameters measured included serum glucose, fasting plasma glucose, and glycated hemoglobin (HbA1c) concentrations.

- Fasting plasma glucose (FPG) levels were measured using an enzymatic colorimetric method with hexokinase, performed on an automated analyzer (e.g., Roche Cobas 8000 (Roche Diagnostics, Rotkreuz, Switzerland) or equivalent). Participants fasted for at least 8–10 h prior to sample collection.
- Serum glucose was assessed via the glucose oxidase–peroxidase (GOD-POD) method, which is a widely accepted and validated enzymatic technique.
- Glycated hemoglobin (HbA1c) levels were determined using high-performance liquid chromatography (HPLC), a standardized method traceable to the Diabetes Control and Complications Trial (DCCT) reference [40].

Statistical Analysis

All statistical procedures were conducted using SPSS version 22.0 (IBM Corp., Armonk, NY, USA). Categorical data were presented as frequencies and percentages (*n*, %), whereas continuous variables were reported as mean ± standard deviation (SD) for normally distributed data, or as median values with interquartile ranges (IQR; 25th–75th percentiles) for non-normally distributed data.

Group comparisons for categorical variables were performed using the Pearson chi-square test. The Kolmogorov–Smirnov test was employed to evaluate the normality of continuous variable distributions. For comparisons between two independent groups, normally distributed variables were analyzed using Student’s *t*-test, while the Mann–Whitney U test was applied for variables that did not meet normality assumptions.

Relationships between continuous variables were examined using Pearson’s correlation analysis for data demonstrating normal distribution, whereas Spearman’s correlation analysis was utilized for variables that deviated from normality.

Furthermore, linear regression analysis was performed to identify predictors of the emotional eating scale subdimensions. The models were built using the Enter method, incorporating variables that showed significant correlations in prior analyses. In addition, a multivariate logistic regression analysis was conducted to evaluate the risk factors associated with GDM. Statistical significance was set at a *p*-value below 0.05 for all tests.

## 3. Results

The research involved 82 pregnant participants, including 37 women diagnosed with GDM forming the case group and 45 healthy pregnant women without GDM serving as the control group. No significant difference was observed in the average age between the two groups (*p* = 0.289).

Evaluation of educational attainment showed that a larger share of the case group had education levels at primary school or below (40.5%) and high school (29.7%), while the control group had a higher proportion of university graduates (51.1%). Despite these differences, the variation between groups was not statistically significant (*p* = 0.082).

No statistically significant differences were identified between the groups in terms of residential location, economic background, employment status, tobacco and alcohol use, or personal and family history of mental illness (*p* > 0.05) (see Table 1).

A notably greater percentage of women in the case group reported having experienced gestational diabetes in prior pregnancies (18.9%) compared to just 2.2% in the control group (*p* = 0.020). Additionally, insulin treatment was only present among the case group participants, with 56.8% receiving therapy, while none of the control group required insulin, indicating a statistically significant difference (*p* < 0.001).

Likewise, the incidence of positive oral glucose tolerance test (OGTT) results was significantly higher in the case group (56.8%), whereas all individuals in the control group exhibited negative OGTT outcomes (*p* < 0.001). In addition, the case group showed notably increased levels of fasting blood glucose (*p* < 0.001), postprandial blood glucose (*p* < 0.001), and HbA1c (*p* = 0.002) when compared to the control group (refer to Table 2).

There were no statistically significant differences between the groups regarding the scores on psychological assessments and eating behavior scales (*p* > 0.05) (refer to Table 3).

Among participants in the case group, the happiness subscale of the Salzburg Emotional Eating Scale (SEES) showed a significant negative correlation with the sadness and anxiety subscales, as well as with obstetric factors such as the number of pregnancies, gravida, parity, and fasting blood glucose levels. Furthermore, the sadness subscale of the SEES was positively correlated with both the anger and anxiety subscales. A positive association was also noted between the anger and anxiety subscales of the SEES. Additionally, scores on the Beck Depression Inventory (BDI) were significantly positively correlated with those on the Beck Anxiety Inventory (BAI) (see Table 4).

In the control group, a significant positive correlation was found between REZZY scores and BMI. A positive correlation was also observed between the Salzburg sadness subscale and both anger and anxiety scores, while sadness scores were negatively correlated with BAI (Beck Anxiety Inventory) and DEBQ (Dutch Eating Behavior Questionnaire) scores.

Similarly, Salzburg anger scores were positively correlated with anxiety but negatively correlated with BDI (Beck Depression Inventory), BAI, and DEBQ scores. Salzburg anxiety scores showed a positive correlation with BMI and negative correlations with both BDI and BAI scores.

Additionally, BDI scores were positively associated with both BAI scores and parity (number of births). (See Table 5).

According to the multiple linear regression analysis, sadness scores are predicted solely by anger (β = 0.640, *p* = 0.041). Anger scores, in turn, are predicted by both anxiety (β = 0.405, *p* = 0.002) and sadness (β = 0.190, *p* = 0.032). Anxiety scores are predicted only by anger (β = 0.598, *p* = 0.003) (Table 6).

In the multivariate logistic regression analysis, psychological and eating behavior assessments did not significantly predict GDM (*p* > 0.05) (Table 7).

## 4. Discussion

This research sought to investigate the connections between anxiety, depression, emotional eating, and eating-disorder-related factors in pregnant women diagnosed with GDM, as well as to compare these variables between women with and without GDM. The results showed that women with GDM experienced mild anxiety and depression levels. Notably, these women tended to reduce their food intake during periods of anxiety, even though they did not fulfill the diagnostic criteria for eating disorders.

As far as we are aware, this study is the first to thoroughly examine the clinical characteristics associated with anxiety, depression, emotional eating, and eating disorders specifically in women diagnosed with gestational diabetes. These results contribute valuable insight into the psychosocial profile of women with GDM and underscore the complex interplay between psychological well-being and eating behaviors during pregnancy.

Several studies have proposed that anxiety and depression may activate the sympathetic adrenal medulla system, stimulate adrenocorticotropic hormone (ACTH) secretion, and elevate levels of glucocorticoids, glucagon, and catecholamines. These hormonal changes can promote gluconeogenesis and glycogen breakdown, ultimately leading to increased blood glucose levels in pregnant women [40]. For instance, a randomized controlled trial demonstrated that women with GDM experiencing anxiety had higher blood glucose levels compared to those without anxiety. Similarly, women with GDM and depression showed elevated blood glucose levels relative to their non-depressed counterparts [41]. Psychological stress in GDM may contribute to impaired glycemic control through activation of the hypothalamic–pituitary–adrenal axis and the sympathetic nervous system, leading to elevated levels of stress hormones. These hormonal changes can also worsen hyperglycemia. Furthermore, anxiety and depression can worsen metabolic regulation by reducing adherence to dietary and treatment recommendations. These mechanisms suggest that they may underlie the association between GDM and increased anxiety or depressive symptoms observed in some studies.

Furthermore, research indicates that pregnant women exhibiting depressive symptoms tend to have poorer glycemic control and higher blood glucose levels. These individuals also often display lower optimism regarding their ability to regulate blood glucose, consistent with findings from other studies [42]. In addition, some investigations have reported significantly higher HbA1c values among pregnant women with GDM who present with anxiety compared to control groups, with positive correlations observed between HbA1c levels and anxiety or depression scores [34,43,44]. It is important to acknowledge that certain studies have identified specific types and severity levels of anxiety as being closely associated with HbA1c concentrations [45,46]. Earlier research has classified pregnant women diagnosed with GDM into groups with high and low anxiety levels using the anxiety subscale of the State-Trait Anxiety Inventory (STAI), which assesses patients’ trait anxiety. These studies found that the high-anxiety group exhibited significantly higher HbA1c levels compared to the low-anxiety group. This phenomenon may be explained by the propensity of individuals with heightened anxiety to demonstrate more pronounced and sustained reactions to daily stressors, thereby experiencing elevated and prolonged stress levels [47]. Increased anxiety and depression in GDM women may lead to poor glycemic control through neuroendocrine mechanisms, including elevated cortisol and catecholamine levels that impair insulin sensitivity. Furthermore, psychological distress may reduce treatment adherence and self-care. The association between anxiety severity and HbA1c levels suggests that mental health assessment would be beneficial to GDM management to optimize metabolic outcomes.

Overall, when considered alongside the recent literature, there is consistent evidence that anxiety and depression during pregnancy negatively impact blood glucose regulation and HbA1c levels in women with GDM. In our study, we observed that pregnant women displayed low levels of anxiety and mild depressive symptoms; however, contrary to expectations, the diagnosis of GDM did not significantly influence these scale scores. This discrepancy may be explained by the relatively small sample size of our cohort. In addition, psychological symptoms may vary according to factors such as individual coping mechanisms, socioeconomic status or social support, which were not assessed in this study. The timing of psychological assessment during pregnancy may have influenced the detection of psychological symptoms, as emotional responses may fluctuate between trimesters. These factors should be considered in future research using larger, longitudinal cohorts.

Although pregnancy represents a significant life event during which women may experience heightened psychological distress and alterations in eating behaviors, limited research has examined the impact of psychological distress on the eating patterns of pregnant women [48]. A 2021 study involving 210 pregnant participants reported that these women experienced moderate levels of stress and emotional eating. While obesity is recognized as a major risk factor for gestational diabetes, the study also found that emotional eating behaviors tended to increase in parallel with rising body mass index (BMI) levels [49].

Furthermore, another study demonstrated significant changes in emotional eating and food consumption between the second and third trimesters of pregnancy. In particular, high depressive symptoms in the second trimester are linked to increased emotional eating and poorer dietary intake in the third trimester. Consequently, depressive symptoms during pregnancy were associated with elevated emotional eating behaviors [50]. This association may be explained by the tendency of women with eating disorders to experience intensified negative emotions upon becoming pregnant, possibly related to pregnancy-associated weight gain and the stress arising from the perceived necessity to eat adequately for fetal development [51]. Although some evidence suggests that women may decrease disordered eating behaviors during pregnancy—motivated by concern for their baby or a new perspective on weight gain—other studies indicate that symptoms and cognitions related to eating disorders remain elevated in this population [52,53]. Eating disorders during the perinatal period have been linked to increased levels of depression and anxiety both during pregnancy and postpartum [54]. In our study, we found that pregnant women tended to eat less when experiencing anxiety, even in the absence of a diagnosed eating disorder. To our knowledge, this is the first study to explore the clinical characteristics related to anxiety, depression, emotional eating, and eating disorders specifically in women diagnosed with gestational diabetes. These findings are consistent with previous research suggesting that disordered eating behaviors in pregnant women may emerge in the preclinical period in response to psychological distress, even in the absence of a formal eating disorder diagnosis. The tendency to reduce food intake under anxiety may reflect a restrictive coping mechanism distinct from the more commonly observed emotional overload.

The limitations of our study include its single-center design, reliance on self-report scales, and relatively small sample size. The use of self-report questionnaires may introduce response bias as respondents may under- or over-report symptoms due to social desirability or recall inaccuracies. Nonetheless, despite these constraints, we believe that our findings provide valuable contributions to the literature by being among the first to examine eating disorders, emotional eating, depression, and anxiety symptoms in women diagnosed with gestational diabetes.

## 5. Conclusions

This study examined the relationship between GDM and psychological symptoms and eating behavior patterns in pregnant women. Although no statistically significant differences were observed in psychiatric symptom severity and eating behavior scores between GDM and control groups, individuals with GDM scored numerically higher in multiple psychological and behavioral domains. These findings may indicate a possible subclinical vulnerability in those with gestational diabetes, especially during the physiologically and emotionally challenging period of pregnancy, which may not be captured by standard diagnostic thresholds. The results suggest that it may be important to integrate psychological screening into the routine management of GDM, even in the absence of overt psychopathology. Early recognition of mental disorders or maladaptive eating patterns may facilitate timely intervention and reduce the risk of poor metabolic and perinatal outcomes. A multidisciplinary approach involving obstetricians, endocrinologists, dietitians and mental health professionals may positively impact both physical and psychological outcomes in this high-risk group, given the metabolic regulation and emotional well-being in GDM. Furthermore, given the increasing prevalence of GDM worldwide and the potential long-term health consequences for both mother and offspring, future studies with larger sample sizes and longitudinal designs are needed to better elucidate causal relationships and evaluate the effectiveness of integrated care models that address both metabolic and mental health needs. Comprehensively addressing these factors may contribute to improved maternal quality of life as well as better infant health outcomes.

## Figures and Tables

**Table 1:** Comparison of the sociodemographic characteristics of the groups.

		GDM Group		Control Group		*p*
		*n*	%	*n*	%	
Age, Avr ± SD		31.7 ± 6.1		30.3 ± 5.8		0.289 *
Education	Primary education and below	15	40.5	16	35.6	0.082 **
	High school	11	29.7	6	13.3	
	University	11	29.7	23	51.1	
Type of settlement	Rural	7	18.9	6	13.3	0.491 **
	Urban	30	81.1	39	86.7	
Economic status	Low	9	24.3	8	17.8	0.467 **
	Medium	28	75.7	37	82.2	
Employment status	Employment	8	21.6	14	31.1	0.334 **
	Nonemployment	29	78.4	31	68.9	
Smoking status	Present	6	16.2	3	6.7	0.287 **
	Absent	31	83.8	42	93.3	
Alcohol use	Present	3	8.1	0	0.0	0.088 **
	Absent	34	91.9	45	100.0	
Known history of mental illness	Present	1	2.7	3	6.7	0.623 **
	Absent	36	97.3	42	93.3	
Family history of mental illness	Present	4	10.8	3	6.7	0.695 **
	Absent	33	89.2	42	93.3	

* Chi-square test, ** Mann–Whitney’s U test.

**Table 2:** Comparison of pregnancy and diabetes characteristics between the groups.

		GDM Group		Control Group		*p*
		*n*	%	*n*	%	
Gestational period	2nd Trimester	5	13.5	7	15.6	0.795 *
	3rd Trimester	32	86.5	38	84.4	
History of gestational diabetes in previous pregnancy	Present	7	18.9	1	2.2	0.020 *
	Absent	30	81.1	44	97.8	
Regular obstetric follow-up	Present	36	97.3	42	93.3	0.623 *
	Absent	1	2.7	3	6.7	
Use of insulin	Present	21	56.8	0	0.0	<0.001 *
	Absent	16	43.2	45	100.0	
Oral glucose tolerance test (OGTT)	Positive	21	56.8	45	100.0	<0.001 *
	Negative	16	43.2	0	0.0	
Number of pregnancies, Median (IQR)		2.0 (1.0–4.0)		2.0 (1.0–3.0)		0.190 **
Gravida, Median (IQR)		2.0 (1.0–4.0)		2.0 (1.0–3.0)		0.367 **
Parity, Median (IQR)		1.0 (0.0–2.0)		0.0 (0.0–2.0)		0.289 **
BMI, Mean ± SD		30.4 ± 4.4		29.3 ± 4.9		0.307 ***
FPG, Mean ± SD		100.3 ± 18.7		78.1 ± 7.9		<0.001 ***
PPG, Mean ± SD		150.0 ± 41.4		103.7 ± 17.5		<0.001 ***
HbA1c, Median (IQR) (Hemoglobin A1c)		5.5 (5.1–6.0)		5.1 (4.9–5.4)		0.002 **

* Chi-square test, ** Mann–Whitney’s U test, *** Student’s *t*-test were applied.

**Table 3:** Comparison of scale scores between groups.

	GDM Group	Control Group	*p*
	Median (IQR)	Median (IQR)	
REZZY	0.0 (0.00–1.0)	0.0 (0.0–1.0)	0.252
SEES Happiness	3.0 (3.0–3.4)	3.0 (3.0–3.4)	0.791
SEES Sadness	2.8 (2.0–3.0)	2.6 (2.2–3.0)	0.817
SEES Anger	2.6 (2.2–3.0)	2.6 (2.0–3.0)	0.873
SEES Anxiety	2.2 (2.0–3.0)	2.0 (1.8–3.0)	0.615
BDI, Mean ± SD	9.4 ± 5.3	8.7 ± 5.3	0.585
BAI, Mean ± SD	14.1 ± 9.2	11.7 ± 7.5	0.193

**Table 4:** Correlation of scale scores in the case group.

		REZZY	Happiness	Sadness	Anger	Anxiety	BDI	BAI
Happiness	r	0.003						
	*p*	0.987						
Sadness	r	−0.145	−0.426					
	*p*	0.391	0.009					
Anger	r	0.003	−0.240	0.539				
	*p*	0.986	0.152	0.001				
Anxiety	r	0.016	−0.355	0.577	0.580			
	*p*	0.925	0.031	0.000	0.000			
BDI	r	0.067	0.217	−0.130	−0.007	−0.221		
	*p*	0.694	0.196	0.442	0.968	0.189		
BAI	r	0.266	0.207	−0.047	0.217	−0.093	0.364	
	*p*	0.111	0.219	0.780	0.197	0.582	0.027	
Age	r	0.099	−0.063	−0.178	0.102	0.085	−0.012	−0.206
	*p*	0.561	0.711	0.292	0.547	0.617	0.942	0.222
Number of pregnancies	r	0157	−0.410	0.001	0.080	0.160	−0.066	0.006
	*p*	0.354	0.012	0.996	0.636	0.343	0.700	0.971
Gravida	r	0.174	−0.456	0.000	0.085	0.106	−0.121	0.014
	*p*	0.311	0.005	0.998	0.623	0.540	0.482	0.933
Parity	r	0.196	−0.359	−0.115	−0.116	−0.036	−0.111	−0.018
	*p*	0.259	0.034	0.512	0.508	0.836	0.526	0.918
BMI	r	0.037	−0.215	0.289	0.217	−0.062	−0.321	0.052
	*p*	0.830	0.201	0.083	0.196	0.714	0.053	0.762
FPG	r	0.065	−0.331	0.074	0.026	0.043	0.003	0.095
	*p*	0.704	0.045	0.664	0.878	0.803	0.984	0.578
PPG	r	0.156	0.092	−0.148	−0.287	−0.242	0.150	0.203
	*p*	0.358	0.589	0.380	0.085	0.150	0.375	0.228
HbA1c	r	−0.072	−0.085	−0.090	0.066	0.006	−0.099	0.093
	*p*	0.670	0.615	0.596	0.698	0.971	0.561	0.584

**Table 5:** Linear regression analysis of factors associated with Salzburg Stress Eating Scale.

		REZZY	Happiness	Sadness	Anger	Anxiety	BDI	BAI
Happiness	r	−0.043						
	*p*	0.781						
Sadness	r	0.175	0.109					
	*p*	0.251	0.478					
Anger	r	0.165	−0.069	0.730				
	*p*	0.279	0.652	0.000				
Anxiety	r	0.153	−0.077	0.718	0.809			
	*p*	0.315	0.615	0.000	0.000			
BDI	r	0.039	−0.009	−0.153	−0.327	−0.346		
	*p*	0.799	0.954	0.316	0.028	0.020		
BAI	r	0.188	−0.146	−0.372	−0.363	−0.414	0.506	
	*p*	0.216	0.340	0.012	0.014	0.005	0.000	
Age	r	−0.149	0.011	−0.034	0.060	0.164	0.050	−0.187
	*p*	0.329	0.943	0.824	0.697	0.283	0.743	0.220
Number of pregnancies	r	−0.015	−0.165	−0.010	0.081	0.016	0.058	−0.169
	*p*	0.924	0.280	0.947	0.596	0.917	0.704	0.266
Gravity	r	−0.010	−0.162	0.014	0.055	0.033	0.060	−0.170
	*p*	0.949	0.307	0.932	0.728	0.835	0.706	0.282
Parity	r	0.086	−0.108	0.051	0.013	−0.038	0.367	0.000
	*p*	0.590	0.494	0.748	0.935	0.813	0.017	0.999
BMI	r	0.425	−0.054	0.225	0.205	0.419	−0.088	−0.122
	*p*	0.004	0.726	0.137	0.177	0.004	0.565	0.425
FBG	r	−0.123	−0.262	−0.247	−0.066	0.041	−0.119	−0.025
	*p*	0.422	0.082	0.103	0.669	0.787	0.438	0.870
PPG	r	−0.061	0.134	−0.309	−0.403	−0.240	0.196	0.092
	*p*	0.691	0.379	0.039	0.006	0.113	0.198	0.547
HbA1c	r	−0.147	−0.169	−0.259	−0.049	−0.015	0.040	0.104
	*p*	0.335	0.268	0.086	0.749	0.921	0.796	0.498

**Table 6:** Linear regression analysis of factors associated with Salzburg Stress Eating Scale.

	β	SE	Standard β	t	*p*
Happiness (R^2^ = 0.081; F = 1.495; *p* = 0.239)					
Sadness	−0.139	0.112	−0.227	−1.243	0.222
Anxiety	−0.086	0.160	−0.099	−0.539	0.593
Sadness (R^2^ = 0.318; F = 5.138; *p* = 0.005)					
Anger	0.640	0.302	0.385	2.120	0.042
Anxiety	0.245	0.261	0.171	0.940	0.354
Happiness	−0.263	0.241	−0.161	−1.089	0.284
Anger (R^2^ = 0.449; F = 13.859; *p* < 0.001)					
Anxiety	0.405	0.122	0.469	3.316	0.002
Sadness	0.190	0.085	0.316	2.232	0.032
Anxiety (R^2^ = 0.391; F = 7.063; *p* = 0.001)					
Happiness	−0.062	0.161	−0.055	−0.387	0.701
Sadness	0.106	0.113	0.153	0.940	0.354
Anger	0.598	0.185	0.517	3.241	0.003

**Table 7:** Logistic regression analysis of the presence of GDM.

	B	*p*	Av	%95 GA
REZZY	0.212	0.418	1.236	0.740–2.065
Salzburg Happiness	0.296	0.465	1.345	0.607–2.978
Salzburg Sadness	0.200	0.562	1.221	0.622–2.399
Salzburg Anger	−0.097	0.843	0.908	0.348–2.364
Salzburg Anxiety	0.121	0.787	1.128	0.470–2.711
BDI	−0.003	0.948	0.997	0.902–1.101
BAI	0.037	0.260	1.038	0.973–1.107

## Data Availability

The original contributions presented in this study are included in the article. Further inquiries can be directed to the corresponding author.
